# Evaluation of a Rapid Point-of-Care Multiplex Immunochromatographic Assay for the Diagnosis of Enteric Fever

**DOI:** 10.1128/mSphere.00253-20

**Published:** 2020-06-10

**Authors:** Shailendra Kumar, Ariana Nodoushani, Farhana Khanam, Alyssa T. DeCruz, Paul Lambotte, Robert Scott, Isaac I. Bogoch, Krista Vaidya, Stephen B. Calderwood, Taufiqur R. Bhuiyan, Javan Esfandiari, Edward T. Ryan, Firdausi Qadri, Jason R. Andrews, Richelle C. Charles

**Affiliations:** a Chembio Diagnostic Systems, Inc., Medford, New York, USA; b Division of Infectious Diseases, Massachusetts General Hospital, Boston, Massachusetts, USA; c International Centre for Diarrhoeal Disease Research, Bangladesh (icddr,b), Dhaka, Bangladesh; d Department of Medicine, University of Toronto, Ontario, Canada; e Dhulikhel Hospital, Dhulikhel, Nepal; f Department of Medicine, Harvard Medical School, Boston, Massachusetts, USA; g Department of Microbiology, Harvard Medical School, Boston, Massachusetts, USA; h Department of Immunology and Infectious Diseases, Harvard T. H. Chan School of Public Health, Boston, Massachusetts, USA; i Division of Infectious Diseases and Geographic Medicine, Stanford University School of Medicine, Stanford, California, USA; University of Missouri-Kansas City School of Medicine

**Keywords:** *S*. Paratyphi A, *S*. Typhi, *Salmonella*, diagnostic, enteric fever, paratyphoid, point-of-care, typhoid

## Abstract

There is a critical need for an improved rapid diagnostic for enteric fever. We have previously demonstrated that serum IgA responses targeting Salmonella enterica serovar Typhi hemolysin E (HlyE) and lipopolysaccharide (LPS) are able to discriminate patients with acute typhoid from healthy controls in areas where enteric fever is endemic (healthy endemic controls) and from patients with other bacterial infections. We now have data demonstrating that IgA antibody responses against these antigens also work well for identifying patients with acute *S.* Paratyphi A infection. To develop a test for acute enteric fever detection, we have adapted a point-of-care immunochromatographic dual-path platform technology (DPP), which improves on the traditional lateral flow technology by using separate sample and conjugate paths and a compact, portable reader, resulting in diagnostics with higher sensitivity and multiplexing abilities. In this analysis, we have compared our standard enzyme-linked immunosorbent assay (ELISA) method to the DPP method in detecting acute phase plasma/serum anti-HlyE and anti-LPS IgA antibodies in a cohort of patients with culture-confirmed *S.* Typhi (*n* = 30) and Paratyphi A infection (*n* = 20), healthy endemic controls (*n* = 25), and febrile endemic controls (*n* = 25). We found that the DPP measurements highly correlated with ELISA results, and both antigens had an area under the curve (AUC) of 0.98 (sensitivity of 92%, specificity of 94%) with all controls and an AUC of 0.98 (sensitivity of 90%, specificity of 96%) with febrile endemic controls. Our results suggest that the point-of-care DPP Typhoid System has high diagnostic accuracy for the rapid detection of enteric fever and warrants further evaluation.

**IMPORTANCE** Enteric fever remains a significant global problem, and control programs are significantly limited by the lack of an optimal assay for identifying individuals with acute infection. This is especially critical considering the recently released World Health Organization (WHO) position paper endorsing the role of the typhoid conjugate vaccine in communities where enteric fever is endemic. A reliable diagnostic test is needed to assess and evaluate typhoid intervention strategies and determine which high-burden areas may benefit most from a vaccine intervention. Our collaborative team has developed and evaluated a point-of-care serodiagnostic assay based on detection of anti-HlyE and LPS IgA. Our finding of the high diagnostic accuracy of the DPP Typhoid System for the rapid detection of enteric fever has the potential to have significant public health impact by allowing for improved surveillance and for control and prevention programs in areas with limited laboratory capacity.

## Abstract (precis)

Enteric fever remains a significant global problem, and control programs are significantly limited by the lack of an optimal assay for identifying individuals with acute infection. This is especially critical considering the recently released World Health Organization (WHO) position paper endorsing the role of the typhoid conjugate vaccine in communities where enteric fever is endemic. A reliable diagnostic test is needed to assess and evaluate typhoid intervention strategies and determine which high-burden areas may benefit most from a vaccine intervention. Our collaborative team has developed and evaluated a point-of-care serodiagnostic assay based on detection of anti-HlyE and LPS IgA. Our finding of the high diagnostic accuracy of the DPP Typhoid System for the rapid detection of enteric fever has the potential to have significant public health impact by allowing for improved surveillance and for control and prevention programs in areas with limited laboratory capacity.

## INTRODUCTION

Typhoid and paratyphoid fever, collectively known as enteric fever, affect more than 14 million people globally and result in around 135,000 deaths each year (1). Enteric fever is prevalent in low-and-middle-income countries (LMICs) that lack access to clean drinking water and improved sanitation, especially in southeast Asia, south Asia, and sub-Saharan Africa (1).

A major unresolved issue in the management, prevention, and control of enteric fever is the absence of a reliable and rapid diagnostic assay. Clinical diagnosis is unreliable (2), and the current reference standard, blood culture, has several limitations, including a low sensitivity of approximately 52 to 70% (3), a several day lag between sample collection and result availability, and requirement of substantial laboratory capacity. Serum-based diagnostics such as the Widal agglutination test, and commercially available assays such as Typhidot and Tubex, while simple and rapid, offer only moderate accuracy in specificity and sensitivity (4). Detecting antibodies secreted from circulating, activated lymphocytes (TPTest) has high sensitivity and specificity for diagnosing enteric fever, but it requires moderately advanced laboratory capacity and requires 18 to 48 h to obtain results (5–7). A rapid diagnostic for enteric fever could improve medical management, reduce overdiagnosis and overuse of antityphoid antimicrobials, which has driven antimicrobial resistance, and improve disease burden estimates and surveillance in areas where enteric fever is endemic, so that informed decisions can be made surrounding vaccine introduction (5). This is especially relevant given the WHO’s prequalification and endorsement of the typhoid-conjugate vaccine (TCV) (8).

There have been several high-throughput immunoscreens of the Salmonella enterica serovar Typhi proteome to identify promising antigens that can be used to develop a serodiagnostic assay that allows for accurate identification of patients with enteric fever (9–13). The top candidate antigens have included *S.* Typhi lipopolysaccharide (LPS), hemolysin E (HlyE), cytolethal distending toxin B (CdtB), flagellin, outer membrane protein A (OmpA), pathogenicity island effector proteins SipB and SipC, among others (9–13). All these studies have identified antibody responses to LPS and/or HlyE among the best discriminators of acute typhoid patients from healthy controls from areas where enteric fever is endemic (endemic healthy controls) and other febrile controls (9–13). In a recent analysis, we applied supervising learning methods and two independent cohorts from Bangladesh and Nepal to identify the best antigen and antibody isotype combinations to identify patients with acute typhoid fever. We found that serum IgA responses targeting *S*. Typhi hemolysin E (HlyE) and LPS are able to discriminate patients with acute typhoid illness from healthy endemic area controls as well as from patients with other bacterial infections (14). We now have data demonstrating that IgA antibody responses against these antigens also work well for identifying patients with acute *S.* Paratyphi A infection, which accounts for 10 to 50% of enteric fever infections in areas of Asia (1, 15).

**FIG 1:**
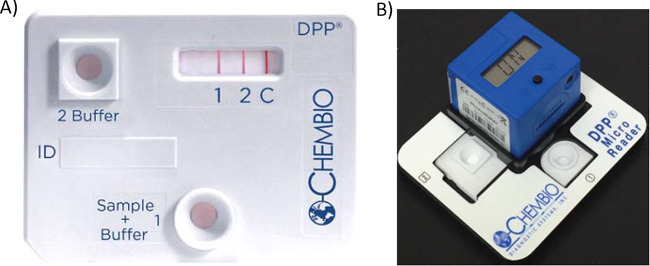
DPP Typhoid System. The test cassette and DPP Micro Reader with holder and typhoid test device are shown. (A and B) The DPP Typhoid System consists of a test cassette, which consist of a sample path and reagent path which intersect in the analyte detection area labeled 1 (LPS), 2 (HlyE), and C (control) (A) and the DPP Micro Reader, a portable, battery-powered instrument that displays a numerical intensity value for the test lines (B).

To translate the serologic testing of HlyE and LPS IgA responses into a multiplex rapid test for enteric fever, we have used Chembio’s patented DPP (Dual Path Platform [16, 17]), a point-of-care immunochromatographic technology. The DPP Typhoid System consists of a sample path that distributes a small volume of sample (∼10 μl of whole blood, plasma, or serum) onto an antibody detection strip containing a test line for LPS, a test line for HlyE, and a control line (Fig. 1). Results are obtained with the DPP Micro Reader, a portable, battery-powered instrument using assay-specific algorithms to verify the presence of the control line and displays a numerical intensity value for each test line. The device has been designed to minimize human interpretation error. This multiplex system has the capability of measuring plasma IgA responses to both LPS and HlyE with high sensitivity and specificity, and its results were highly correlated with ELISA results.

## RESULTS AND DISCUSSION

### Characterization of anti-LPS and HlyE IgA responses.

We evaluated plasma and serum IgA responses to LPS and HlyE antigens by ELISA and DPP Typhoid System using previously collected samples from three cohorts of individuals: (i) patients at the acute phase of enteric fever (day of presentation to a health facility), with blood culture-confirmed *S*. Typhi (*n* = 30) or *S*. Paratyphi A (*n* = 20); (ii) healthy controls from Bangladesh, an area where typhoid is endemic (*n* = 25); and (iii) febrile controls from Nepal with other bacteremias (i.e., Staphylococcus aureus, Escherichia coli, Klebsiella pneumoniae, *Streptococcus* spp.; *n* = 25).

**FIG 2:**
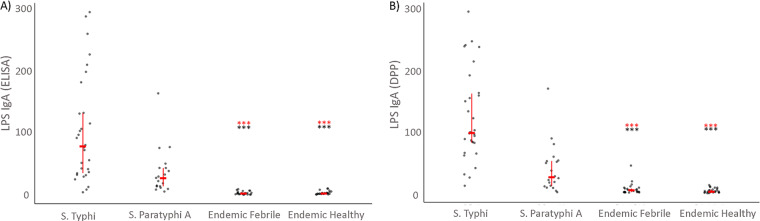
Characterization of anti-LPS IgA plasma responses using ELISA (A) and the DPP Typhoid System (B). Individual and median anti-LPS responses with interquartile range for patients at acute phase (day 0) of enteric fever (*S.* Typhi or Paratyphi A), healthy and febrile controls from a typhoid-endemic area (endemic healthy and endemic febrile). Differences between cases and control groups were assessed using the Mann-Whitney test. A *P* of <0.05 was considered significant. ***, *P* < 0.0001 (red, *S*. Typhi; black, *S*. Paratyphi A).

**FIG 3:**
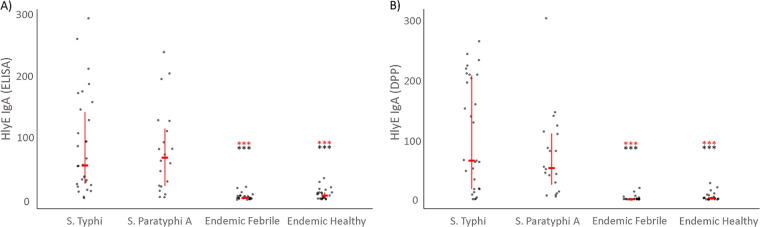
Characterization of anti-HlyE IgA plasma responses using ELISA (A) and DPP (B). Individual and median anti-HlyE responses with interquartile range for patients at acute phase (day 0) of enteric fever (*S.* Typhi or Paratyphi A), healthy and febrile controls from a typhoid-endemic area (endemic healthy and endemic febrile). Differences between cases and control groups were assessed using the Mann-Whitney test. A *P* of <0.05 was considered significant. ***, *P* < 0.0001 (red, *S*. Typhi; black, *S*. Paratyphi A).

We found higher IgA immunoreactivity to LPS and HlyE in *S*. Typhi and *S*. Paratyphi A cases by ELISA and DPP compared to healthy controls from areas where enteric fever is endemic (endemic healthy controls) (*P* < 0.0001) and endemic febrile controls (*P* < 0.0001) (Fig. 2 and 3, respectively).

### Comparison of DPP Typhoid System measurements to reference ELISA results.

**FIG 4:**
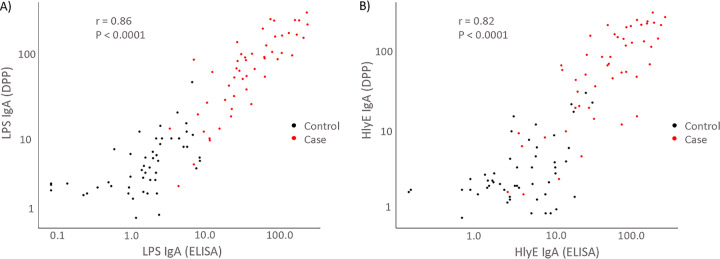
Correlation between ELISA and DPP Typhoid System measurements. (A and B) Plot of anti-LPS (A) and anti-HlyE (B) IgA plasma measurements by ELISA versus DPP Typhoid System of acute enteric fever cases (red, *S.* Typhi or *S.* Paratyphi A) and controls (black, endemic healthy and febrile controls). The Pearson correlation coefficient (*r*) is shown.

**FIG 5:**
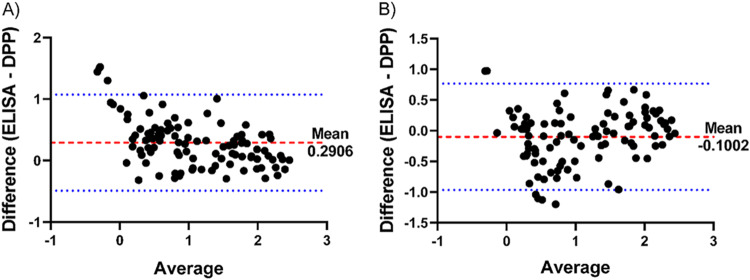
Bland-Altman plot of ELISA and DPP Typhoid System measurements. (A and B) Plot of log-transformed anti-LPS (A) and anti-HlyE (B) IgA plasma measurements by ELISA and DPP Typhoid System of acute enteric fever (*S.* Typhi and *S*. Paratyphi A) cases and controls (endemic healthy and febrile controls). The red dashed line indicates the mean difference between measurements. The blue dotted lines represent the 95% limits of agreement.

The anti-LPS and HlyE IgA ELISA and DPP measurements had a high degree of linear correlation in both negative and positive serum samples, *r* = 0.86 (*P* < 0.0001) and *r* = 0.82 (*P* < 0.0001), respectively (Fig. 4). To further characterize DPP performance agreement with ELISA, we also performed a Bland-Altman plot of the log-transformed data, which demonstrated strong agreement between the two tests without significant bias (Fig. 5).

### Accuracy of the DPP Typhoid System.

**FIG 6:**
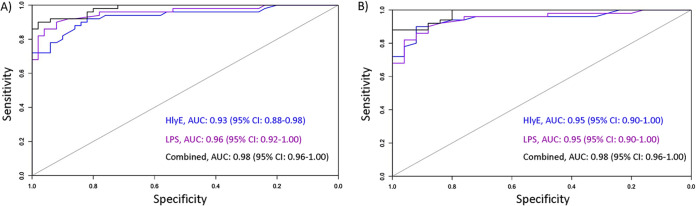
Receiver operating characteristic (ROC) curve for anti-LPS and HlyE IgA response using DPP for the diagnosis of acute enteric fever. ROC curve plotting the specificity versus sensitivity of distinguishing patients with acute enteric fever (*S*. Typhi or *S.* Paratyphi A) from all endemic controls (healthy and febrile controls) (A) and endemic febrile controls (B). 95% CI, 95% confidence interval.

We assessed classification accuracy of the DPP Typhoid System by receiver operating characteristic (ROC) area under the curve (AUC) (Table 1 and Fig. 6). The combined antigens (HlyE and LPS) in the DPP Typhoid System distinguished plasma from enteric fever cases from individuals presenting with other invasive bacteremias with a sensitivity of 90% and specificity of 96% (AUC, 0.98). HlyE alone had an AUC of 0.95 (sensitivity of 90%, specificity of 92%), and LPS alone had an AUC of 0.95 (sensitivity of 90%, specificity of 88%). When including all endemic controls (healthy and febrile), the DPP had an AUC of 0.98 for the combined antigens (sensitivity of 92%, specificity of 94%), AUC of 0.93 for HlyE alone (sensitivity of 90%, specificity of 84%) and AUC of 0.96 for LPS alone (sensitivity of 90%, specificity of 92%).

**TABLE 1:** Receiver operating characteristic area under the curve (AUC) for anti-HlyE and LPS IgA using DPP for distinguishing enteric fever cases (*S.* Typhi or *S.* Paratyphi A) patients from controls^*a*^

Antigen(s)	Febrile controls			All endemic controls		
	AUC	Specificity (%)^*b*^	Sensitivity (%)^*c*^	AUC	Specificity (%)	Sensitivity (%)
HlyE	0.95 (0.90−1.00)	92	89	0.93 (0.88−0.98)	84	84
LPS	0.95 (0.90−1.00)	88	88	0.96 (0.92−1.00)	92	91
**Both**	**0.98 (0.9−1.00)**	**96**	**90**	**0.98 (0.96−1.00)**	**98**	**92**

a Values for both antigens are shown in boldface type.

b Specificity at 90% sensitivity.

c Sensitivity at 90% specificity.

We also evaluated receiver operating characteristic curves for *S*. Typhi and *S.* Paratyphi A cases, independently, for each antigen (see Table S1 in the supplemental material). Use of the combined antigens (HlyE or LPS) by the DPP Typhoid System could discriminate typhoid fever from endemic febrile controls with a sensitivity of 96% and specificity of 100% (AUC, 1.00), and all endemic controls with a sensitivity of 96% and specificity of 100% (AUC, 1.00). These results were in line with our prior findings of excellent discriminatory values for HlyE and LPS IgA responses in identifying patients with typhoid fever. The discriminatory power of anti-LPS IgA for paratyphoid A fever and controls was not as high as for typhoid fever, but the combined antigens measured by DPP could accurately distinguish samples from patients with paratyphoid fever from samples of endemic febrile controls with a sensitivity of 90% and specificity of 92% (AUC, 0.97) and from all endemic controls with a sensitivity of 90% and specificity of 88% (AUC, 0.97) (Table S1).

10.1128/mSphere.00253-20.1 TABLE S1 Receiver operating characteristic (ROC) area under the curve (AUC) for anti-HlyE and LPS IgA using DPP for distinguishing *S.* Typhi or *S.* Paratyphi A patients from controls. Download Table S1, DOCX file, 0.1 MB. Copyright © 2020 Kumar et al. 2020 Kumar et al. This content is distributed under the terms of the Creative Commons Attribution 4.0 International license.

### Conclusion.

There is a critical need for a rapid, accurate diagnostic assay for enteric fever. Available rapid serodiagnostics for typhoid fever have moderate sensitivity and specificity (3), and there have been limited evaluations of rapid test diagnostics for paratyphoid fever (4). In this study, we developed and evaluated a rapid assay for the detection of patients with typhoid and paratyphoid fever, the DPP Typhoid System, based on the detection of serum/plasma IgA responses to LPS and HlyE. To evaluate the performance of the assay, we tested samples collected from enteric fever cases and controls by the DPP Typhoid System and by our reference ELISA method (14). The detection of anti-HlyE and LPS IgA in samples by DPP showed excellent agreement with results from ELISA, demonstrated high sensitivity and specificity in identifying patients with culture-confirmed enteric fever, and distinguished those individuals from controls in areas where enteric fever is endemic. These data suggest that the DPP Typhoid System may be a promising tool for diagnosing individuals with enteric fever.

Advantages of this potential point-of-care assay include its ease of handling, rapid turnaround time, minimal sample volume requirement (with potential to use 10 μl of finger prick blood, serum, or plasma), and minimal to no requirement of laboratory capacity or training. In addition, it can identify individuals with either *S*. Typhi or *S*. Paratyphi A infection. Prior serodiagnostic assays for typhoid fever have been based on IgG and/or IgM responses to various *S*. Typhi target antigens (4). These assays have had limited sensitivity and specificity due to the high background seroprevalence of IgG and the cross-reactivity of IgM in areas where enteric fever is endemic (4). Measurement of IgA in the DPP Typhoid System overcomes some of these limitations due to the relative transience in plasma of antigen-specific IgA compared to IgG antibodies and improved specificity over IgM antibodies (11).

A limitation of our analysis is that the test thus far has been evaluated only with stored plasma and serum samples from a small cohort of adult cases and controls from Asia. Future studies will need to include assessment of assay performance for (i) other sample types (e.g., capillary whole blood from finger stick); (ii) other populations/settings (e.g., Africa, elsewhere in Asia); (iii) different age groups, particularly young children; (iv) different stages of illness (e.g., days of fever prior to presentation), and (v) various alternative febrile illness (e.g., dengue, chikungunya, scrub typhus, and other invasive bacteremias, particularly invasive nontyphoidal *Salmonella* [iNTS]), to evaluate for cross-reactive antibodies to HlyE and LPS. HlyE is present in human-specific *Salmonella* serovars, *S*. Typhi and *S*. Paratyphi A. Although absent in the primary *Salmonella* serovars causing iNTS (*S.* Typhimurium and *S.* Enteritidis), it can be found in other iNTS (including *S.* Schwarzengrund, Montevideo, Bredeney) (18–20) as well as several strains of E. coli (21, 22). We have previously demonstrated that HlyE IgA retained discriminatory value with other non-*Salmonella* Gram-negative organisms (i.e., E. coli and *Klebsiella*) (14). Another study of Nigerian children that included 86 *S*. Typhi and 29 iNTS culture-confirmed cases demonstrated negligible IgA responses to HlyE except for two patients with low immunoreactivity (11). This study did demonstrate some cross-reactivity to *S.* Typhi LPS in patients with iNTS (IgM > IgG > IgA) (11), which may potentially be due to conserved epitopes in the core lipopolysaccharide and lipid A regions (11). Further studies of DPP will need to be performed to investigate whether IgA responses to our selected antigens (LPS and HlyE) are able to discriminate enteric fever from iNTS.

Despite the limitations of our analysis, the excellent agreement of the DPP Typhoid System with our ELISA method, which has been tested more broadly (14), suggests that the DPP Typhoid System is a promising assay for the rapid detection of enteric fever and warrants further prospective analysis.

## MATERIALS AND METHODS

### Plasma/serum samples.

Samples were obtained from participants with enteric fever on the day of presentation to the International Centre for Diarrheal Disease Research, Dhaka, Bangladesh (icddr,b) Dhaka hospital, Mirpur field site (Dhaka, Bangladesh), or Dhulikhel Hospital, a Kathmandu University Hospital (Kavrepalanchowk), Nepal, with self-reported fever of 3- to 7-day duration without an obvious focus of infection or alternate diagnosis. Bacteremia was confirmed by blood culture using BacT/Alert or Bactec 9050 automated system (BD Diagnostics) with identification of isolates by standard culture and biochemical tests (23, 24). Serum/plasma was also collected from healthy typhoid-endemic and North American controls and from North American patients presenting with an alternative febrile illness (PCR-confirmed influenza or bacteremia with S. aureus, E. coli, or K. pneumoniae). All samples were collected with the approval of the following Research and Ethical Review Committees and/or Institutional Review Board for Human Subjects Research: International Centre for Diarrheal Disease Research, Dhaka, Bangladesh (icddr,b), Nepal Health Research Council (Kathmandu, Nepal), Stanford University, and Massachusetts General Hospital. Written informed consent was obtained from all individuals or their guardians prior to study participation. Additional North American healthy control samples used in this study were procured from ZeptoMetrix, Franklin, MA, USA.

### Enzyme-linked immunosorbent assay (ELISA).

ELISA was performed on all the samples using *S*. Typhi LPS and purified HlyE as previously described (14). To compare across plates, we normalized ELISA results by calculating the ratio of each sample reading to that of a standard included on the same plate. The standard for HlyE was a chimeric monoclonal antibody to HlyE and for LPS, it was an in-house serum pool made from sera from patients with typhoid fever. The ratio was multiplied by 100 and expressed as ELISA units.

### Development of chimeric HlyE MAbs.

Immunizations and hybridoma development were performed by the Monoclonal Antibody Core facility of the Dana-Farber Cancer Institute, Boston, MA, as previously described (25, 26). Briefly, three mice (BALB/c, C57BL/6, and Swiss-Webster), 4 to 6 weeks old, were obtained from Charles River Laboratories (Wilmington, MA). All animals were acquired and maintained according to the guidelines of the Institutional Animal Care and Use Committee of Harvard Standing Committee. Mice were immunized at three subcutaneous sites and one intraperitoneal site with 50 μg of purified HlyE emulsified with an equal volume of complete Freund’s adjuvant (Sigma Chemical Co., St. Louis, MO). Mice were again boosted at day 14, and sera were collected. The mouse with the highest serum titer to HlyE was boosted again at day 35, and then the spleen and lymph nodes were collected, and cells were processed for fusion with SP 2/0 myeloma cells (ATCC no. CRL8-006, Rockville, MD) at a ratio of 2:1. Positive hybridomas and subclones were selected by indirect ELISA on HlyE and counterscreened with an irrelevant antigen. The sequenced variable regions of the light and heavy chains of selected hybridomas were cloned into pcDNA3.4 vector containing the human IgG1 and IgA1 heavy chain. The resulting plasmids were then transfected into Expi293F cells. Antibodies were purified from the cell culture supernatant by IgA affinity resin and analyzed by sodium dodecyl sulfate-polyacrylamide gel electrophoresis (SDS-PAGE), Western blotting, and size exclusion chromatography (SEC)-high-performance liquid chromatography (HPLC) (TSKgel G3000SWxl column; Tosho) to confirm molecular weight and purity. Sequencing, cloning, and antibody purification were performed by GenScript (Piscataway, NJ).

### DPP Typhoid System.

The DPP Typhoid System employs Chembio’s patented Dual Path Platform (DPP) technology, which consists of a sample path and reagent path that intersect in the analyte detection area in the readout window of the test cassette that is labeled test (1, 2) and control (C) (Fig. 1). It employs an antibody conjugated (anti-human IgA) to colloidal gold dye particles and *S.* Typhi LPS and HlyE antigens that are bound to the membrane for capture of the antibody, if present in the sample. To initiate the test, a 10-μl specimen is diluted with 5 drops (150 μl) of sample buffer in a sample tube, and 100 μl sample and buffer mixture is applied to the Sample + Buffer well (well 1) of the DPP test cassette. The specimen flows along the sample path membrane and is delivered to the test zone of the reagent strip, where specific LPS and HlyE antigens (test 1 and 2, respectively) and a control (C) (protein A) are immobilized. If the specimen contains anti-LPS anti-HlyE IgA antibodies, they bind instantly to the respective immobilized test antigens (test 1 and 2), while nonspecific antibody binds to the protein A control (C) line. Five minutes after adding the specimen, 6 drops (150 μl) of buffer are added to the Buffer well (well 2). The buffer hydrates and releases the anti-human IgA antibody gold conjugate, which migrates to the test zone and binds to the captured IgA antibodies targeting LPS and/or HlyE in the respective test areas, producing a pink/purple line. The gold conjugate continues to migrate through the membrane, producing a pink/purple line in the control (C) area containing protein A. This procedural control serves to demonstrate that specimen and reagents have been properly applied and have migrated through the device. In the absence of antibodies against LPS or HlyE in the patient’s sample, there are no pink/purple lines produced in the test 1 and 2 area.

Results were read using the DPP Micro Reader (Fig. 1) between 15 and 20 min after the addition of the sample/buffer to well 1. The DPP Micro Reader is a portable, battery-powered instrument that records the reflectance of the test strip surface and uses assay-specific algorithms to interpret the color intensity of the control and test lines. It displays a qualitative result for each analyte (reactive, nonreactive, or invalid) after approximately 3 s based on test-specific cutoff values loaded into the reader. The reader also displays numerical values of test line intensity, allowing for semiquantitative evaluation of antibody levels.

### Statistical analysis.

The distribution of antibody responses in culture-confirmed cases and controls was compared by Wilcoxon rank sum test. The agreement between ELISA and DPP measurements was assessed by Pearson’s correlation and Bland-Altman analysis. The accuracy of ELISA and DPP were assessed by receiver operator characteristic area under the curve (ROC AUC). All analyses were performed using GraphPad Prism 8.2.0 and R software version 3.6.0 (R Project for Statistical Computing; https://www.R-project.org/).

## References

[1] GBD Typhoid and Paratyphoid Collaborators. 2019 The global burden of typhoid and paratyphoid fevers: a systematic analysis for the Global Burden of Disease Study 2017. Lancet Infect Dis 19:369–381. doi:10.1016/S1473-3099(18)30685-6. 30792131 PMC6437314

[2] Andrews JR, Barkume C, Yu AT, Saha SK, Qamar FN, Garrett D, Luby SP 2018 Integrating facility-based surveillance with healthcare utilization surveys to estimate enteric fever incidence: methods and challenges. J Infect Dis 218:S268–S276. doi:10.1093/infdis/jiy494. 30184162 PMC6226762

[3] Mogasale V, Ramani E, Mogasale VV, Park J 2016 What proportion of Salmonella Typhi cases are detected by blood culture? A systematic literature review. Ann Clin Microbiol Antimicrob 15:32. doi:10.1186/s12941-016-0147-z. 27188991 PMC4869319

[4] Wijedoru L, Mallett S, Parry CM 2017 Rapid diagnostic tests for typhoid and paratyphoid (enteric) fever. Cochrane Database Syst Rev 5:CD008892. doi:10.1002/14651858.CD008892.pub2. 28545155 PMC5458098

[5] Andrews JR, Ryan ET 2015 Diagnostics for invasive Salmonella infections: current challenges and future directions. Vaccine 33(Suppl 3):C8–C15. doi:10.1016/j.vaccine.2015.02.030. 25937611 PMC4469564

[6] Islam K, Sayeed MA, Hossen E, Khanam F, Charles RC, Andrews J, Ryan ET, Qadri F 2016 Comparison of the performance of the TPTest, Tubex, Typhidot and Widal immunodiagnostic assays and blood cultures in detecting patients with typhoid fever in Bangladesh, including using a Bayesian latent class modeling approach. PLoS Negl Trop Dis 10:e0004558. doi:10.1371/journal.pntd.0004558. 27058877 PMC4825986

[7] Khanam F, Sheikh A, Sayeed MA, Bhuiyan MS, Choudhury FK, Salma U, Pervin S, Sultana T, Ahmed D, Goswami D, Hossain ML, Mamun KZ, Charles RC, Brooks WA, Calderwood SB, Cravioto A, Ryan ET, Qadri F 2013 Evaluation of a typhoid/paratyphoid diagnostic assay (TPTest) detecting anti-Salmonella IgA in secretions of peripheral blood lymphocytes in patients in Dhaka, Bangladesh. PLoS Negl Trop Dis 7:e2316. doi:10.1371/journal.pntd.0002316. 23951368 PMC3708850

[8] World Health Organization. 2019 Typhoid vaccines: WHO position paper, March 2018 - Recommendations. Vaccine 37:214–216. doi:10.1016/j.vaccine.2018.04.022. 29661581

[9] Charles RC, Liang L, Khanam F, Sayeed MA, Hung C, Leung DT, Baker S, Ludwig A, Harris JB, Larocque RC, Calderwood SB, Qadri F, Felgner PL, Ryan ET 2014 Immunoproteomic analysis of antibody in lymphocyte supernatant in patients with typhoid fever in Bangladesh. Clin Vaccine Immunol 21:280–285. doi:10.1128/CVI.00661-13. 24371257 PMC3957676

[10] Charles RC, Sheikh A, Krastins B, Harris JB, Bhuiyan MS, LaRocque RC, Logvinenko T, Sarracino DA, Kudva IT, Eisenstein J, Podolsky MJ, Kalsy A, Brooks WA, Ludwig A, John M, Calderwood SB, Qadri F, Ryan ET 2010 Characterization of anti-Salmonella enterica serotype Typhi antibody responses in bacteremic Bangladeshi patients by an immunoaffinity proteomics-based technology. Clin Vaccine Immunol 17:1188–1195. doi:10.1128/CVI.00104-10. 20573880 PMC2916242

[11] Darton TC, Baker S, Randall A, Dongol S, Karkey A, Voysey M, Carter MJ, Jones C, Trappl K, Pablo J, Hung C, Teng A, Shandling A, Le T, Walker C, Molina D, Andrews J, Arjyal A, Basnyat B, Pollard AJ, Blohmke CJ 2017 Identification of novel serodiagnostic signatures of typhoid fever using a Salmonella proteome array. Front Microbiol 8:1794. doi:10.3389/fmicb.2017.01794. 28970824 PMC5609549

[12] Davies DH, Jain A, Nakajima R, Liang L, Jasinskis A, Supnet M, Felgner PL, Teng A, Pablo J, Molina DM, Obaro SK 2016 Serodiagnosis of acute typhoid fever in Nigerian pediatric cases by detection of serum IgA and IgG against hemolysin E and lipopolysaccharide. Am J Trop Med Hyg 95:431–439. doi:10.4269/ajtmh.15-0869. 27215295 PMC4973195

[13] Liang L, Juarez S, Nga TV, Dunstan S, Nakajima-Sasaki R, Davies DH, McSorley S, Baker S, Felgner PL 2013 Immune profiling with a Salmonella Typhi antigen microarray identifies new diagnostic biomarkers of human typhoid. Sci Rep 3:1043. doi:10.1038/srep01043. 23304434 PMC3540400

[14] Andrews JR, Khanam F, Rahman N, Hossain M, Bogoch II, Vaidya K, Kelly M, Calderwood SB, Bhuiyan TR, Ryan ET, Qadri F, Charles RC 2018 Plasma IgA responses against two Salmonella Typhi antigens identify patients with typhoid fever. Clin Infect Dis 68:949–955. doi:10.1093/cid/ciy578. PMC6399438 30020426

[15] Arndt MB, Mosites EM, Tian M, Forouzanfar MH, Mokhdad AH, Meller M, Ochiai RL, Walson JL 2014 Estimating the burden of paratyphoid A in Asia and Africa. PLoS Negl Trop Dis 8:e2925. doi:10.1371/journal.pntd.0002925. 24901439 PMC4046978

[16] Chembio Diagnostic Systems Inc. 3 2007. Dual path immunoassay device. US patent 7189522.

[17] Chembio Diagnostic Systems Inc. 9 2006. Dual path immunoassay device. International patent WO/2006/099191.

[18] den Bakker HC, Moreno Switt AI, Govoni G, Cummings CA, Ranieri ML, Degoricija L, Hoelzer K, Rodriguez-Rivera LD, Brown S, Bolchacova E, Furtado MR, Wiedmann M 2011 Genome sequencing reveals diversification of virulence factor content and possible host adaptation in distinct subpopulations of Salmonella enterica. BMC Genomics 12:425. doi:10.1186/1471-2164-12-425. 21859443 PMC3176500

[19] Fuentes JA, Villagra N, Castillo-Ruiz M, Mora GC 2008 The Salmonella Typhi hlyE gene plays a role in invasion of cultured epithelial cells and its functional transfer to S. Typhimurium promotes deep organ infection in mice. Res Microbiol 159:279–287. doi:10.1016/j.resmic.2008.02.006. 18434098

[20] Suez J, Porwollik S, Dagan A, Marzel A, Schorr YI, Desai PT, Agmon V, McClelland M, Rahav G, Gal-Mor O 2013 Virulence gene profiling and pathogenicity characterization of non-typhoidal Salmonella accounted for invasive disease in humans. PLoS One 8:e58449. doi:10.1371/journal.pone.0058449. 23505508 PMC3591323

[21] Ludwig A, von Rhein C, Bauer S, Huttinger C, Goebel W 2004 Molecular analysis of cytolysin A (ClyA) in pathogenic Escherichia coli strains. J Bacteriol 186:5311–5320. doi:10.1128/JB.186.16.5311-5320.2004. 15292132 PMC490944

[22] Oscarsson J, Westermark M, Lofdahl S, Olsen B, Palmgren H, Mizunoe Y, Wai SN, Uhlin BE 2002 Characterization of a pore-forming cytotoxin expressed by Salmonella enterica serovars typhi and paratyphi A. Infect Immun 70:5759–5769. doi:10.1128/iai.70.10.5759-5769.2002. 12228306 PMC128311

[23] Talawadekar NN, Vadher PJ, Antani DU, Kale VV, Kamat SA 1989 Chloramphenicol resistant Salmonella species isolated between 1978 and 1987. J Postgrad Med 35:79–82. 2621666

[24] Cruickshank R, Duguid JP, Marmion BP, Swain RHA (ed). 1975 The Enterobacteriaceae, Salmonella, p 403–419. *In* Medical microbiology, 12th ed Churchill Livingstone, Edinburgh, UK.

[25] Kearney JF, Radbruch A, Liesegang B, Rajewsky K 1979 A new mouse myeloma cell line that has lost immunoglobulin expression but permits the construction of antibody-secreting hybrid cell lines. J Immunol 123:1548–1550. 113458

[26] Kohler G, Milstein C 1975 Continuous cultures of fused cells secreting antibody of predefined specificity. Nature 256:495–497. doi:10.1038/256495a0. 1172191

