# Supplementary material for: Evaluation of a Rapid Point-of-Care Multiplex Immunochromatographic Assay for the Diagnosis of Enteric Fever
Source: mSphere. 2020 Jun 10;5(3):e00253-20. doi: 10.1128/mSphere.00253-20 (PMC7289704; doi:10.1128/mSphere.00253-20)
Supplement: TABLE S1 [file mSphere.00253-20-st001.docx]

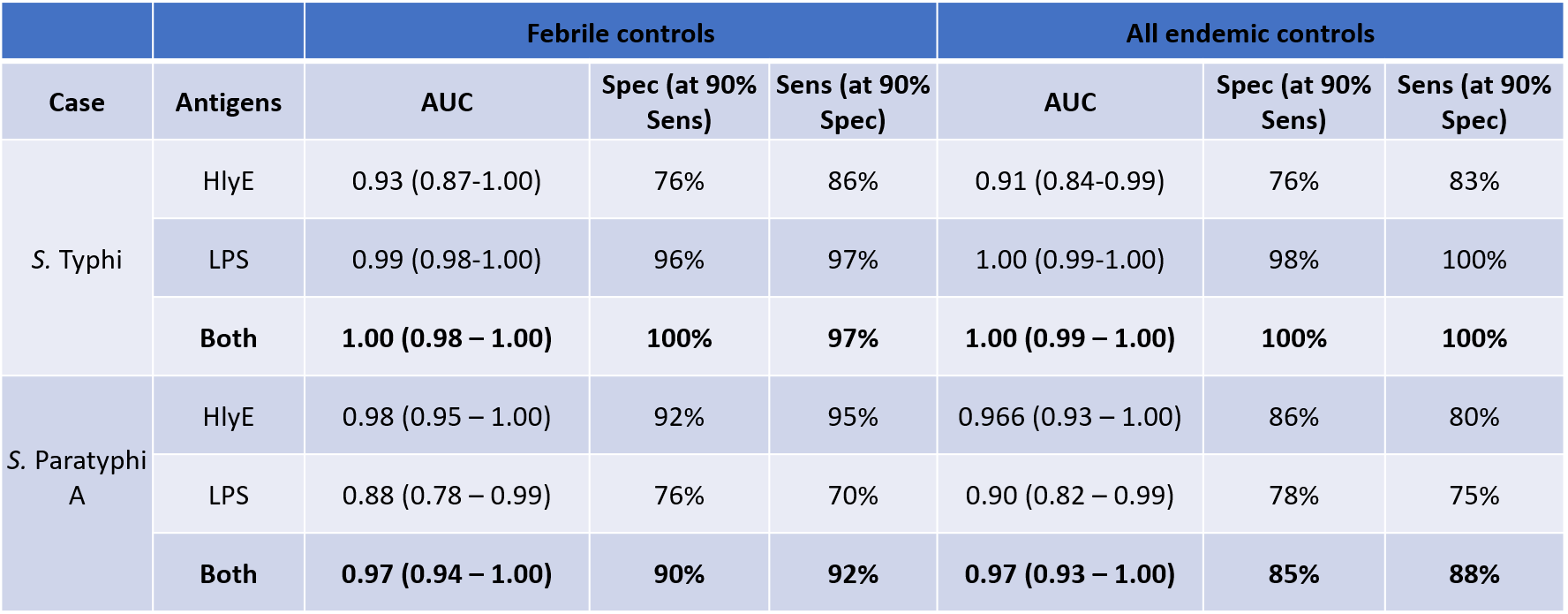


**Table S1: Receiver operating characteristic (ROC) area under the curve (AUC) for anti-HlyE and LPS IgA using DPP for distinguishing *S.* Typhi or *S.* Paratyphi A patients from controls.**
